# Initialized decadal prediction for transition to positive phase of the Interdecadal Pacific Oscillation

**DOI:** 10.1038/ncomms11718

**Published:** 2016-06-02

**Authors:** Gerald A. Meehl, Aixue Hu, Haiyan Teng

**Affiliations:** 1 National Center for Atmospheric Research, 3090 Center Green Drive, Boulder, Colorado 80301, USA

## Abstract

The negative phase of the Interdecadal Pacific Oscillation (IPO), a dominant mode of multi-decadal variability of sea surface temperatures (SSTs) in the Pacific, contributed to the reduced rate of global surface temperature warming in the early 2000s. A proposed mechanism for IPO multidecadal variability indicates that the presence of decadal timescale upper ocean heat content in the off-equatorial western tropical Pacific can provide conditions for an interannual El Niño/Southern Oscillation event to trigger a transition of tropical Pacific SSTs to the opposite IPO phase. Here we show that a decadal prediction initialized in 2013 simulates predicted Niño3.4 SSTs that have qualitatively tracked the observations through 2015. The year three to seven average prediction (2015–2019) from the 2013 initial state shows a transition to the positive phase of the IPO from the previous negative phase and a resumption of larger rates of global warming over the 2013–2022 period consistent with a positive IPO phase.

## Abstract (web summary)

The phase of the Interdecadal Pacific Oscillation (IPO) impacts global temperatures. Here, the authors show that a build-up of off-equatorial western tropical Pacific heat content allows ENSO events to trigger IPO phase changes, with a predicted switch to a positive IPO for 2015–2019 and larger warming trends.

## Graphical abstract

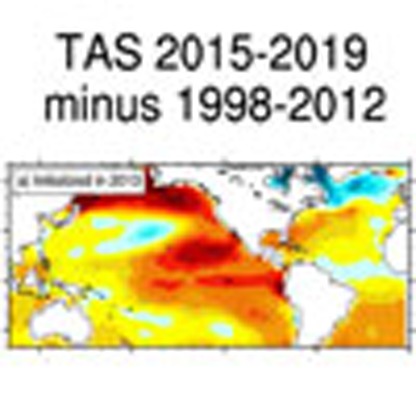

Retrospective hindcasts have shown skill in simulating the two phase changes of the Interdecadal Pacific Oscillation (IPO) since 1960, the first from negative to positive in the 1970s and the second from positive to negative in the late 1990s^1^,^2^,^3^,^4^,^5^. A proposed mechanism for IPO multidecadal variability indicates that for such decadal transitions to occur, there should be a build-up of upper ocean heat content in the off-equatorial western tropical Pacific such that an El Niño/Southern Oscillation (ENSO) event can then trigger a transition of tropical Pacific SSTs to the opposite IPO phase^5^,^6^,^7^,^8^.

This mechanism is a variant of the delayed-action oscillator^9^ that depends on off-equatorial wind forcing^10^,^11^, which could be produced by tropical-midlatitude interaction. Convective heating anomalies in the tropical Pacific, associated with precipitation and SST anomalies there, produce anomalous Rossby waves in the atmosphere and sea level pressure signals in the mid-latitude North and South Pacific. These sea level pressure anomalies are associated with surface wind stress curl anomalies that force anomalous ocean Rossby waves near 25°N and 25°S. These slow-moving ocean Rossby waves subsequently produce, on decadal timescales, off-equatorial ocean heat content anomalies in the western tropical Pacific. The build-up of off-equatorial heat content that is necessary for an IPO transition contributes to a 15- to 20-year timescale for the IPO^6^. The interannual variability associated with ENSO, combined with the off-equatorial ocean heat content anomalies at the western boundary, then produce equatorial ocean Kelvin waves that change the equatorial thermocline depth in such a way as to reverse the sign of equatorial Pacific SST anomalies to the opposite phase of the IPO. The mechanism, as originally formulated^6^, has been independently confirmed^7^ and generalized in the context of the subtropical gyre and the Pacific subtropical cells (STCs)^12^. This is a variant of a previous mechanism for Pacific decadal variability that postulated IPO-like variability could be associated with amplitude modulation of ENSO events, thus also connecting interannual and decadal timescale variability in the Pacific^13^.

Transitions in the sign of the IPO have been shown to be simulated in single and multi-model initialized decadal hindcasts and verified both through epoch differences and empirical orthogonal function metrics^1^,^2^,^3^,^4^,^5^. For the negative IPO from roughly 2000–2013, there was a slowdown or reduced rate of global surface warming (also sometimes referred to as the early-2000s hiatus^14^,^15^,^16^,^17^). The previous positive phase of the IPO from the 1970s to late 1990s was associated with an accelerated rate of global warming and has been documented in climate models^17^,^18^,^19^.

Here we connect these transitions to the mechanism described above^6^,^7^ and use those results to provide the context for a decadal prediction initialized in 2013, which shows a transition of the IPO from its recent negative state back to positive. Although there have been earlier attempts to predict a recent IPO transition from negative to positive^20^, here we provide a process-based mechanism on which to base the credibility of the prediction of the transition to the positive phase of the IPO. We choose the CCSM4, because it is the only current climate model with an extensive documentation with regards to processes and initialized predictions involved with IPO transitions^1^,^2^,^17^, as well as initialized predictions of decadal climate variability in the North Atlantic^21^ (see Methods). There are ten ensemble members run for each initial year.

## Results

### Off-equatorial heat content

**Figure 1:**
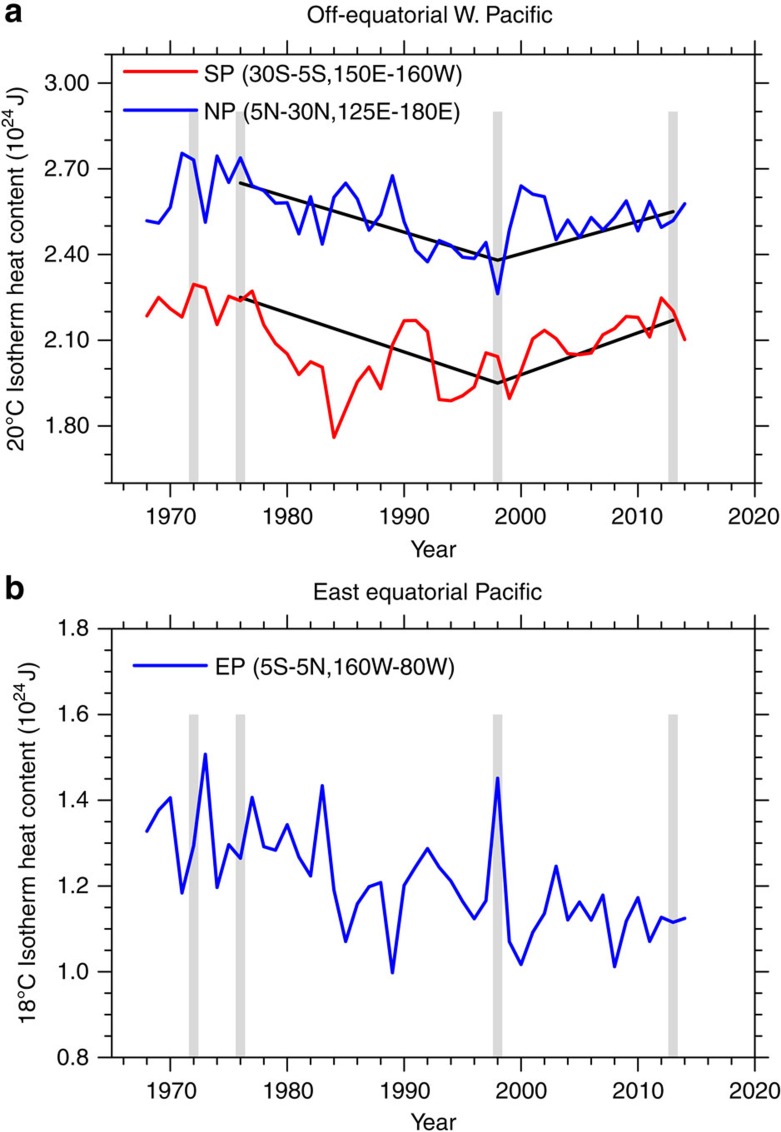
Off-equatorial western Pacific and east equatorial Pacific heat content. (**a**) Annual mean time series of vertically integrated ocean heat content (× 10^24^ J) to the depth of the 20 °C isotherm for regions in the off-equatorial western Pacific from the ocean initial states used for the initialized model hindcasts; blue line for an area in the northwest tropical Pacific (5°N–30°N, 125°E–180°E); red line for an area in the southwest tropical Pacific (5°S–30°S, 150°E–160°W); shaded grey vertical lines indicate start points for the 1972–73 El Niño, 1976–77 El Niño, 1998–2000 La Niña and 2014–2015 El Niño, respectively; solid lines are linear trends from 1976 to 1998 and from 1998 to 2013; (**b**) same as in **a**, except for vertically integrated ocean heat content down to the depth of the 18 °C isotherm for an area in the equatorial eastern Pacific (5°N–5°S, 160°W–80°W).

As noted above, there are two crucial elements of the IPO mechanism outlined above^6^: a build-up of off-equatorial ocean heat content anomalies in the western tropical Pacific over at least a 15-year period before a transition and an interannual ENSO event that triggers a transition (El Niño for transition from negative to positive and La Niña for transition from positive to negative). For the IPO transitions during the period of the CMIP5 decadal hindcasts from 1960 to the early 2000s^22^, the ocean states used to initialize the hindcasts for CCSM4 are shown in Fig. 1. For the tropical northwestern Pacific region (5°N–30°N, 125°E–180°E), the vertically integrated heat content to the depth of the 20 °C isotherm in the ocean initial state is ∼2.7 × 10^24^ J just before the 1976–77 El Niño event, with roughly 2.2 × 10^24^ J in the tropical southwestern Pacific region (5°S–30°S, 150°E–160°W) (Fig. 1a). The 1972–73 El Niño, evidenced by an increase in vertically integrated heat content to the depth of the 18 °C isotherm in the eastern equatorial Pacific (5°S–5°N, 160°W–80°W) to values near 1.5 × 10^24^ J (Fig. 1b) is followed by a subsequent decrease over the next few years in off-equatorial southwestern tropical Pacific heat content by nearly 0.1 × 10^24^ J (Fig. 1a). However, the subsequent 1976–77 El Niño, with an increase in vertically integrated heat content in the eastern equatorial Pacific of ∼1.4 × 10^24^ J (Fig. 1b), is associated with a subsequent drop in off-equatorial northwestern and southwestern tropical Pacific heat content for the next decade and longer (Fig. 1a). With the caveat that the initial ocean state values are less certain before the satellite era that began in the late 1970s (see Methods), this sequence suggests that the 1972–73 El Niño started the discharge of off-equatorial western Pacific heat content that was then given a boost by the 1976–77 El Niño, which closely followed, and thus contributed to the onset of the positive phase of the IPO in the mid-1970s^19^.

Values for the southwestern Pacific region bottom out after the 1982–83 El Niño, which occurred while the IPO was still in a positive phase. The weak La Niña in 1984–85 contributed to a subsequent increase in off-equatorial heat content in the southwestern area of ∼0.3 × 10^24^ J, but the northwestern area heat content continued to decline. Thus, the changes in the southwestern area were necessary but not sufficient to produce an IPO transition and the positive IPO phase continued until the late 1990s.

After the partial recovery around 1990, the southwestern Pacific region heat content declined again until a minimum value of ∼2.0 × 10^24^ J was reached in 1997, whereas there was a minimum value of roughly 2.3 × 10^24^ J in the northwestern Pacific region around 1998, coincident with the large 1997–98 El Niño (Fig. 1a). These low heat content values in the off-equatorial western Pacific in the ocean initial states were then associated with the 2-year La Niña event (1998–2000) with low eastern equatorial Pacific heat content values down to near 1.0 × 10^24^ J (Fig. 1b) and a transition to the negative phase of the IPO. Next, off-equatorial western tropical Pacific vertically integrated heat content values started to climb again until the initial-state heat content in the northwestern tropical Pacific region in 2013 was nearly 2.6 × 10^24^ J, close to its value in the 1976 initial state when the IPO previously transitioned to positive. The southwestern Pacific region heat content in 2012 and 2013 was around 2.1–2.2 × 10^24^ J, also its highest value since the mid-1970s. Indications from these ocean heat content values from the initial states for the initialized hindcasts suggest that if even a moderate El Niño were to occur shortly after 2013, there could be an IPO transition from negative to positive.

It would be tempting to speculate, based on this small sample, that threshold values amounting to around 2.6 × 10^24^ J in the northwestern area and 2.2 × 10^24^ J in the southwestern area would be necessary for an El Niño event to trigger a transition to positive IPO. Conversely, heat content values of roughly 2.4 × 10^24^ and 2.0 × 10^24^ J in the northwestern and southwestern areas, respectively, could be necessary for a La Niña event to trigger a transition to the negative phase of the IPO. However, based on the small sample size and the limitations of the ocean initialization scheme (see Methods), these values can only be considered as instructive at this time, pending further research with more models.

### Hindcast skill

**Figure 2:**
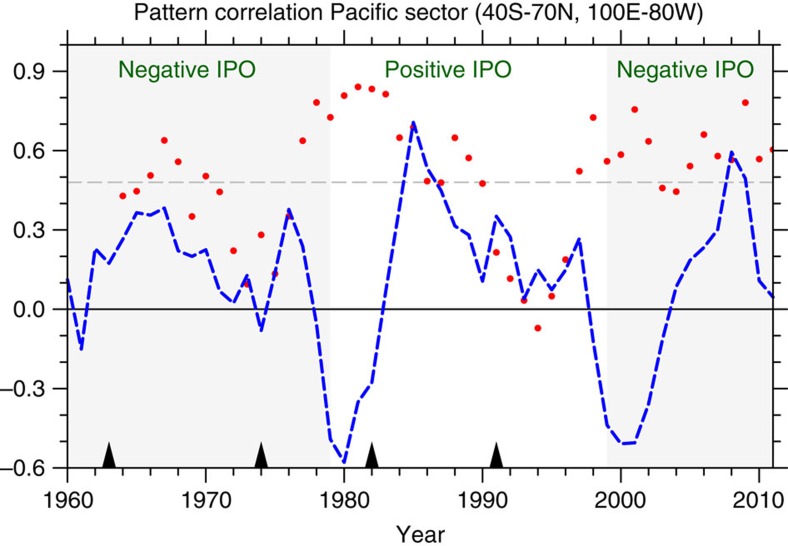
Hindcast skill for the IPO. Anomaly pattern correlation for the Pacific SSTs in hindcasts initialized every year from 1960 for CCSM4 (ocean points only, 40°S–70°N, 100°E–80°W; same as in Fig. 2e in ref. 4, but just for CCSM4 see Methods). Horizontal dashed line is 95% significance. Blue dashed line is persistence prediction defined as the average of years three to seven in the future having the same SST anomalies as the 5-year average before the start of the prediction. Each red dot represents the central year of the 3- to 7-year average hindcast; thus, the value for the initial year 1960 for the prediction averaged for 1962–66 is plotted for 1964 and so on. Phase changes of the IPO from negative to positive in the late 1970s and from positive to negative in the late 1990s are denoted by shading. Hindcast skill notably drops below 95% when influenced by the volcanic eruptions (denoted by triangles above *x* axis) of Fuego in the early 1970s and Pinatubo in the early 1990s, but not Agung in the early 1960s or El Chichon in the early 1980s^23^.

Credibility for an initialized prediction in the future must be evaluated in the context of past predictions or hindcasts. Thus, it is necessary to quantify IPO hindcast skill in CCSM4 to see whether there are false IPO transitions that appear in initialized hindcasts with previous El Niño or La Niña events. Figure 2 shows hindcast verification in terms of pattern correlation for Pacific SSTs (Pacific basin, ocean grid points 40°S–70°N, 100°E–80°W) for CCSM4 for the ensemble average of ten ensemble members for each initial year starting in 1960 (that is, the correlation is calculated using the ensemble average^4^). This is a version of a similar figure shown for initialized hindcasts for all the CMIP5 models^4^. Hindcast skill for the year three to seven predictions (5 year average over years 3–7, hereafter referred to as ‘3- to 7-year' hindcasts or predictions) is defined as a pattern correlation for Pacific SSTs above the 95% significance level (horizontal dashed line, see Methods). Most 3- to 7-year hindcasts are skillful by that measure and exceed persistence (defined as the 5-year average anomalies before the initial year and persisted to forecast years three to seven) except for two periods, one in the mid-1970s and the other in the mid-1990s. These two periods have been shown to have reduced hindcast skill due to the effects of the Fuego and Pinatubo volcanic eruptions, respectively^23^, when the post-eruption sequence of tropical Pacific SSTs does not match the model ensemble-average forced response. The previously documented skill in IPO transitions from negative to positive in the late 1970s and from positive to negative in the late 1990s is denoted by red dots above 95% significance for changes in shading in Fig. 2. Model skill in predicting transitions is indicated by the combination of having a good pattern correlation and poor performance of a persistence forecast (blue dashed line). It is noteworthy that the persistence predictions have no skill in predicting across major decadal transitions (actually having negative values in Fig. 2 at the time of the transitions), while the initialized predictions have significant skill across the transitions. In addition, the CCSM4 shows skill in predicting the correct state of the IPO even for years when there were significant El Niño (for example, no IPO transition for the 1982–83 El Niño) or La Niña (for example, no IPO transition for the 1988–89 La Niña) events.

Figure 2 also addresses the concern that an initialized hindcast could either trend to model climatology and always ‘transition' away from its initial state or simply persist the initial state and never ‘transition' to another IPO state. The performance of the model in the hindcasts shows that, with the exception of the volcano-related drops in skill noted above (with the caveat that the volcanoes affect 11 out of the 48 five-year average prediction periods in Fig. 2), the model can capture IPO transitions as well as persist existing initialized IPO states.

### ENSO as a trigger for decadal transitions

As the previous two IPO transitions were simulated with some success as noted above and if ENSO events act as a trigger for the western tropical Pacific off-equatorial heat content anomalies in the ocean initial states noted in Fig. 1 as in previous studies^6^, then ENSO evolution for those IPO transitions should have been simulated as well. There have been previous indications that ENSO events can be predicted with initialized climate models for lead times of at least 2 years^24^. Here we show the time evolution of the predictions for all ensemble members and the ensemble average for the first 5 years after initialization. Clearly, there will be spread in the predictions, but the hindcast verification in Fig. 2 uses the ensemble average and that is of primary interest here.

**Figure 3:**
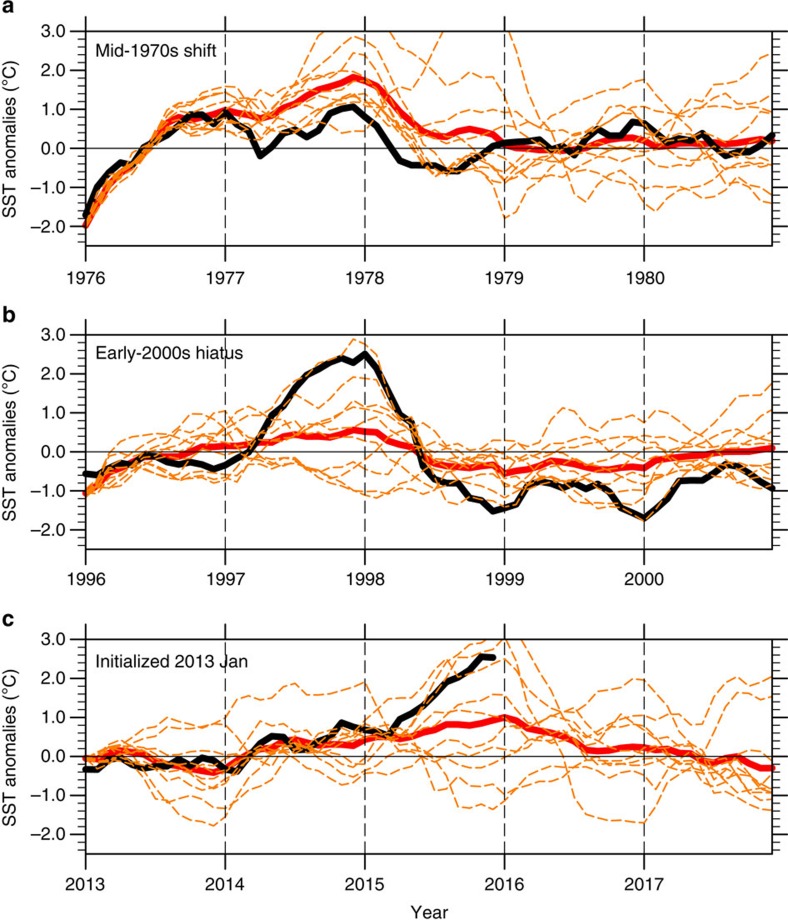
Initialized predictions of eastern equatorial Pacific sea surface temperatures. Monthly mean time series of initialized predictions for the Niño3.4 area (5°S–5°N, 120°W–170°W), calculated as anomalies from a 1975 to 2014 climatology; observations (black lines^34^), individual model ensemble members are dashed orange lines; ensemble average is solid red line; (**a**) prediction for the mid-1970s shift, initialized 1 January 1976 (ref. 1); (**b**) prediction for early-2000s hiatus, initialized 1 January 1996 (ref. 4); (**c**) prediction initialized 1 January 2013.

Owing to the limitations of the ocean initialization scheme that affect ocean initial states more in the pre-satellite (before the late-1970s) than post-satellite era (see Methods), we follow the conservative approach previously used to analyse predictions of the mid-1970s climate shift^1^ and show results for the model initialized in January 1976. The ensemble average for the Niño3.4 index in the eastern equatorial Pacific (5°N–5°S, 170°W–120°W, a standard ENSO index) tracks the observations for the onset of the El Niño in mid-1976 and then the ensemble average remains anomalously warm through 1978, in fact longer than what was observed (Fig. 3a). Although there is spread in the initialized predictions after 1977, the 1976–1977 period of above-normal eastern equatorial SSTs is captured by all ten ensemble members and was associated with the IPO transition from negative to positive for 3- to 7-year predictions during this time period^1^.

For the IPO transition from positive to negative in the late 1990s, there is greater confidence in the ocean initial states (see Methods); thus, we follow a previous analysis^4^ and show the predictions initialized in January 1996 that have more ensemble spread than for the 1976–77 case, but the ensemble average shows above-normal Niño3.4 SSTs for the 1997–98 event of lower amplitude than what was observed. Of more significance for the IPO transition, the ensemble average model prediction shows below-normal Niño3.4 SSTs for the observed 2-year La Niña from 1998 to 2000, with most ensemble members having negative values for that time period in qualitative agreement with the observations. This La Niña simulation was associated with the prediction of the transition of the IPO from positive to negative in the model for the 3- to 7-year predictions initialized in 1996 (ref. 1).

### Prediction for 2015–2019

Thus, there are indications of some model skill in simulating ENSO events that could act as triggers for the off-equatorial ocean heat content in the western tropical Pacific to contribute to IPO transitions (Fig. 1). Therefore, it is of interest to investigate simulations initialized in 2013 for the 3- to 7-year predictions (2015–2019) of the IPO and globally averaged surface air temperature. We choose the initial year 2013 so that we have 2 years of subsequent observations to see whether there has been any skill for predicted Niño3.4 SSTs that could indicate a role in an IPO transition. As noted above, for a credible prediction of an IPO transition, there must not only be heat content build-up in the off-equatorial western Pacific over a period of ∼15 years, but also a reasonable simulation of ENSO for the first few years after initialization, for the 3- to 7-year prediction to have skill.

**Figure 4:**
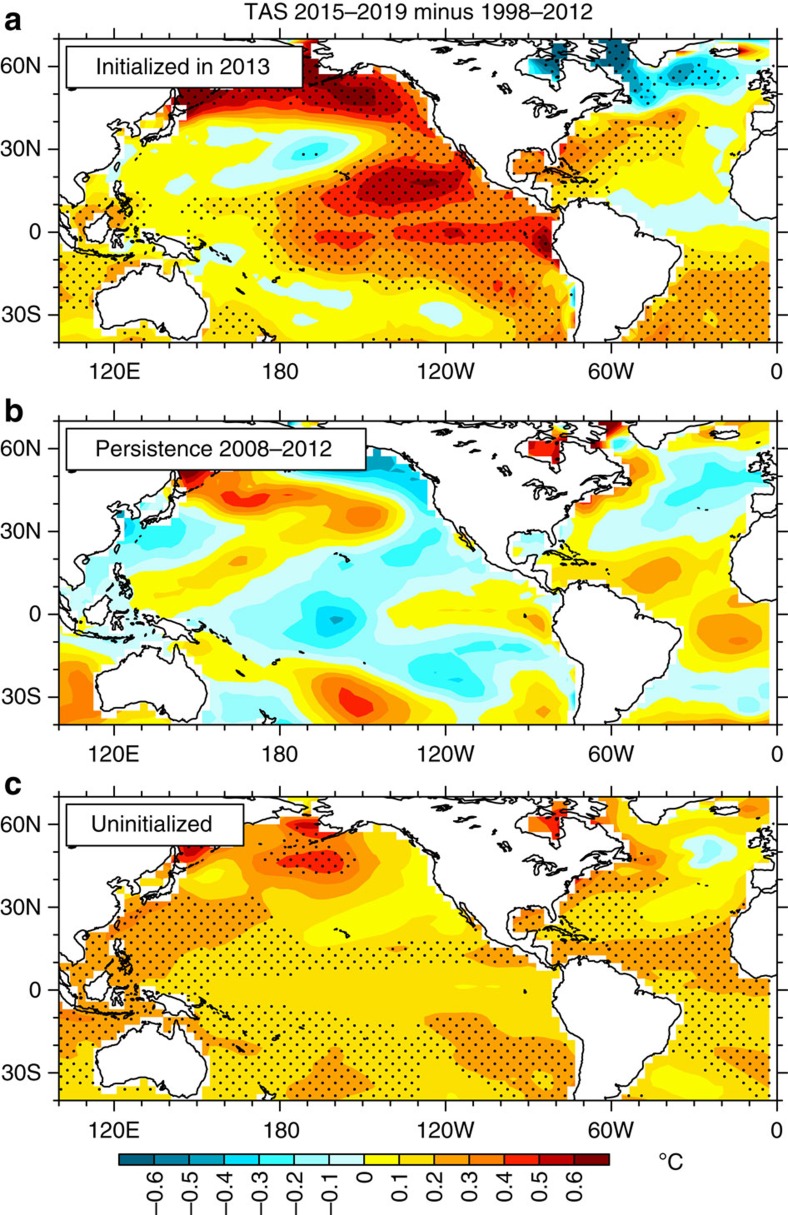
Predicted patterns of surface temperature. (**a**) Global surface air temperature anomaly patterns (°C) for prediction initialized on 1 January 2013 for the 5-year average 2015–2019 minus observed reference period 1998–2012 from NCEP/NCAR reanalyses^34^ (see Methods); (**b**) persistence prediction defined as the average of years three to seven in the future having the same SST anomalies as the 5-year average before the start of the prediction as in Fig. 2, for the 2015–2019 average having the same SST anomalies as the 2008–2012 average, and (**c**) uninitialized prediction for years 2015–2019 minus the model data averaged over the period 1998–2012; stippling indicates 95% significance level from one-sided *t*-test.

For predictions initialized in January 2013 (Fig. 3c), the observations started to warm in 2014 and the model-simulated Niño3.4 SSTs also warmed. In 2015, the observed Niño3.4 SSTs continued to increase, to approach the magnitude of the warmest predicted ensemble members (Fig. 3c). The ensemble average predictions from the model show above-normal Niño3.4 SSTs for 2015 continuing through 2016. The associated SST anomalies for the 3- to 7-year prediction (2015–2019 minus a 15-year average before the initial year, 1998–2012, used as a reference climatology with which to compare the initialized prediction) initialized in 2013 show a positive phase of the IPO, with above-normal SSTs predicted over the eastern tropical Pacific extending into the northeast Pacific, with low amplitude-negative SST anomalies in the northwest and southwest Pacific (Fig. 4a). This pattern is nearly opposite to the persistence prediction (persisting 2008–2012 average anomalies forward to 2015–2019; Fig. 4b) and is also considerably different from the uninitialized simulation that shows warming almost everywhere (Fig. 4c).

**Figure 5:**
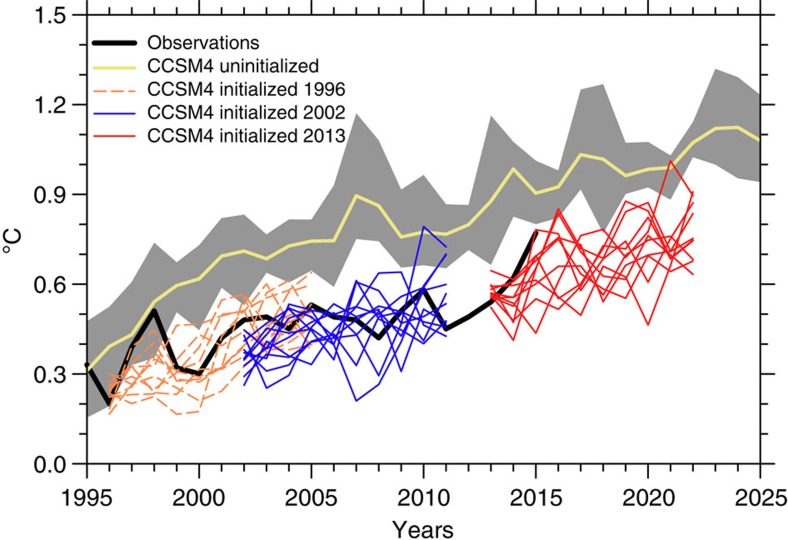
Time series of global surface air temperature anomalies. Annual mean surface temperature anomalies (°C) relative to observed climatology of 1960–1990 for the CCSM4 uninitialized six member ensemble average (solid yellow line) and the envelope of individual ensemble simulations (grey shading); annual mean values from observations is solid black line^25^; individual ensemble member time series for 10-year predictions initialized 1 January 1996 (dashed orange lines) to capture the late-1990s transition from positive to negative IPO^4^, an example of predictions for the hiatus initialized 1 January 2002 (blue lines) and predictions initialized on 1 January 2013 (red lines), the latter showing larger predicted warming rates than the former during the hiatus.

As noted above, there is a strong association between the IPO tendency and globally averaged surface air temperature trends. CCSM4 hindcasts initialized in 1996 simulated the onset of the reduced rate of global surface warming with the transition of the IPO from positive to negative in the late 1990s^4^. The predicted linear trend from 1996 to 2005 of +0.30±0.09 °C per decade (error bars are ±1 s.d.) is smaller than the uninitialized value of +0.39 ±0.08 °C per decade and closer to the observed value of +0.24 °C per decade (Fig. 5). Meanwhile, a hindcast initialized in 2002 during the hiatus has a predicted linear trend from 2002 to 2011 of +0.20±0.11 °C per decade, roughly 30% smaller than the prediction initialized in 1996 before the hiatus.

### Prediction of 2013–2022 global surface temperature trend

With a transition of the IPO from negative to positive in a prediction initialized in 2013, it could be expected from previous studies^14^,^15^,^16^,^17^,^18^ that there should be a resumption of higher rates of globally averaged surface temperature increase. For the prediction initialized in 2013, the linear trend from 2013 to 2022 is +0.22±0.13 °C per decade, nearly 60% larger than the uninitialized projection for that time period of +0.14±0.12 °C per decade (Fig. 5) and nearly three times larger than the observed trend^25^ of +0.08 °C per decade over the period 2001 to 2014 during the early twenty-first century slowdown^26^. This indicates an acceleration of the global surface temperature warming trend over the prediction period from 2013 to 2022 associated with the positive phase of the IPO, compared with that from external forcing alone and compared with the observed warming trend during previous negative phase of the IPO that was associated with a slowdown of the rate of increase of global surface temperature^25^.

## Discussion

The 3- to 7-year hindcasts with CCSM4 initialized every year show skill in simulating patterns of SST in the Pacific associated with the IPO, except for periods when there are disruptions from volcanic eruptions with a post-eruption sequence of Pacific SST patterns that do not agree with the ensemble average forced response in the model^23^. Other studies have shown less skill for the smaller North Pacific area defined for the Pacific Decadal Oscillation (PDO)^27^. Although the PDO and IPO are closely related^28^, the smaller area strictly defined for the PDO in the North Pacific has more spatial noise and less predictive skill for that small area than the much larger Pacific-wide SST pattern defined for the IPO. In addition, the STC in the Southern Hemisphere shows larger variability in some models, affecting the STC transport convergence anomaly that could affect the North Pacific STC and thus the PDO index, which is defined for the North Pacific.

A proposed mechanism for the IPO indicates that a sustained build-up of off-equatorial ocean heat content in the western tropical Pacific for at least 15 years, due to tropical–mid-latitude interaction and ocean Rossby waves associated with the phase of the IPO, is required for an ENSO event to then trigger a transition of the IPO to its opposite phase. Initial state heat content used for the hindcasts with CCSM4 indicates that there was indeed such a build-up before the simulated IPO transition in the mid-1970s that was associated with the prediction of the 1976–77 El Niño event, with possible contributions from the 1972 to 1973 El Niño event. For the late-1990s IPO transition from positive to negative, a heat content deficit in the off-equatorial western tropical Pacific was associated with the simulated 1998–2000 La Niña event. Other El Niño or La Niña events did not trigger an IPO transition, owing to the requisite time for heat content to build up in the off-equatorial western Pacific. Since the late 1990s, heat content has built up in the initial state used for the predictions, thus suggesting that even a moderate-sized El Niño event could trigger a transition to the positive phase of the IPO. Predictions initialized in 2013 show that the simulated Niño3.4 SSTs have tracked the observations with low-amplitude warming in 2014 and larger warming in 2015. Such qualitative success in Niño3.4 simulations for past IPO transitions suggests that an IPO transition probably started in 2014. Indeed, the 3- to 7-year predictions for 2015–2019 that were initialized in 2013 indicate such an IPO transition has occurred, with a resumption of accelerated rates of global warming above those in the uninitialized model simulations. If such predictions were to become operational, the next step (beyond the scope of this study) would be to provide probabilistic climate change information for various time frames in the near-term future.

## Methods

### Initialization procedure

As described in a previous study^21^, the hindcasts in CCSM4 are initialized using a forced ocean–sea ice simulation designed to reproduce the evolution of the ocean and sea ice states from the start of 1948 through the end of 2013. The CCSM4 ocean and sea ice models are coupled and forced at the surface with Coordinated Ocean-ice Reference Experiment (CORE-II) version 2-observed historical atmospheric data^29^,^30^. The forcing data include a complete set of air–sea momentum, heat and freshwater fluxes based on the National Centers for Environmental Prediction/National Center for Atmospheric Research (NCEP/NCAR) reanalyses. There is a weak restoring of model surface salinity to observed climatology and no assimilation of subsurface observations. An integration is run through four consecutive 66-year cycles of 1948–2013 forcing. The ocean and sea ice models in hindcast experiments are initialized with 1 January restart files for a particular year from the last (fourth) cycle of the spin-up simulation. Initial conditions for the atmosphere and land surface are taken from corresponding years of a six-member ensemble of uninitialized twentieth-century runs. The ten-member ensembles run for each initial year starting in 1960 are generated by randomly selecting atmosphere and land initial conditions from different uninitialized twentieth-century runs^31^ and/or from different days in the month of January. Results from these uninitialized twentieth-century experiments^31^ are shown in Figs 4c and 5. The initialized hindcasts and the uninitialized twentieth-century experiments include the same external forcings of solar irradiance, greenhouse gases, aerosols and volcanic activity.

As atmospheric model reanalysis data are used in the CORE-II forcing of the spin-up integrations used for the ocean initial states in the hindcasts, it is likely to be that the quality of the forcing data becomes better in the more recent period, whereas there are issues with reanalysis quality earlier in the record, in particular with precipitation and radiation fluxes over oceans (for example, before the 1970s)^32^,^33^. Therefore, most of the emphasis in this study is on initialized hindcasts after 1975.

### Bias adjustment procedure

The initialized hindcast experiments are bias adjusted to remove model systematic errors^1^,^21^. Climatological monthly mean differences from observations (surface air temperature from the NCEP/NCAR reanalyses)^34^ for the 10-year hindcasts are computed and composited by month following the initial dates of all the hindcast simulations. Next, these average time-evolving monthly mean differences are subtracted from each member of each 10-year hindcast to remove the model systematic error, leaving the model signal from external forcing and internally generated decadal climate variability.

### Assessing hindcast skill

Anomalies for the 3- to 7-year bias-adjusted predictions are calculated as the year 3–7 average minus the average of the observations for the previous 15 years before the initial year of the hindcast^1^,^4^. Other metrics show similar results^2^. To test whether the pattern correlation coefficient between the prediction and observations is distinguishable from chance associations in the large-scale pattern, a Monte Carlo test is performed that consists of 10,000 randomly constructed patterns based on detrended twentieth-century simulations from a multi-model ensemble of CMIP5 models^4^. The 95th percentile of the pattern correlation coefficient of the random pattern is 0.47 in Fig. 2. Persistence is defined as the observed 5-year average before the initial year of the prediction minus a 15-year average for 6–20 years before the initial year^4^.

### Observations

The Nino3.4 index is available from http://www.esrl.noaa.gov/psd/gcos_wgsp/Timeseries/. Observed global mean surface temperature data available from http://www.ncdc.noaa.gov/cag/time-series/global. The NCEP/NCAR reanalyses are available from http://www.esrl.noaa.gov/psd/data/gridded/data.ncep.reanalysis.html.

### Data availability

The CCSM4 model code is available from http://www.cesm.ucar.edu/models/ccsm4.0/index.html. The CCSM4 model data are all available from the Earth System Grid (https://www.earthsystemgrid.org). All analysis codes are available on request.

## Additional information

**How to cite this article:** Meehl, G. A. *et al*. Initialized decadal prediction for transition to positive phase of the Interdecadal Pacific Oscillation. *Nat. Commun.* 7:11718 doi: 10.1038/ncomms11718 (2016).
